# Use of the Stable Nitrogen Isotope to Reveal the Source-Sink Regulation of Nitrogen Uptake and Remobilization during Grain Filling Phase in Maize

**DOI:** 10.1371/journal.pone.0162201

**Published:** 2016-09-08

**Authors:** Lan Yang, Song Guo, Qinwu Chen, Fanjun Chen, Lixing Yuan, Guohua Mi

**Affiliations:** 1 Center for Resources, Environment and Food Security, College of Resources and Environmental Science, China Agricultural University, Beijing, P.R. China; 2 Soil and Fertilizer Research Institute Sichuan Academy of Agricultural Science, Chengdu, P.R. China; Institute of Genetics and Developmental Biology Chinese Academy of Sciences, CHINA

## Abstract

Although the remobilization of vegetative nitrogen (N) and post-silking N both contribute to grain N in maize (*Zea mays* L.), their regulation by grain sink strength is poorly understood. Here we use ^15^N labeling to analyze the dynamic behaviors of both pre- and post-silking N in relation to source and sink manipulation in maize plants. The results showed that the remobilization of pre-silking N started immediately after silking and the remobilized pre-silking N had a greater contribution to grain N during early grain filling, with post-silking N importance increasing during the later filling stage. The amount of post-silking N uptake was largely driven by post-silking dry matter accumulation in both grain as well as vegetative organs. Prevention of pollination during silking had less effect on post-silking N uptake, as a consequence of compensatory growth of stems, husk + cob and roots. Also, leaves continuously export N even though grain sink was removed. The remobilization efficiency of N in the leaf and stem increased with increasing grain yield (hence N requirement). It is suggested that the remobilization of N in the leaf is controlled by sink strength but not the leaf *per se*. Enhancing post-silking N uptake rather than N remobilization is more likely to increase grain N accumulation.

## Introduction

Although nitrogen (N) fertilization is a powerful tool for increasing crop yield, associated N loss from a cropping system can be a major source of environmental pollution [1]. In China, for example, from 1977 to 2005, the total annual grains production increased by 71%while synthetic N fertilizer application increased by 271%. Since the 1990s, such low N use efficiency has contributed to severe environmental problems [2]. Because population growth along with increasing consumption of high-calorie and meat-intensive diets are expected to roughly double human food demand by 2050 [3], the breeding of high-yielding genotypes that use N efficiently is an urgent priority [4].

The relationship between cereal yield and N use has been widely studied [5]. In maize (*Zea mays* L.), two processes contribute to grain N content: remobilization of vegetative N and post-silking N uptake [5–8]. In general, about 35 to 55% of grain N is derived from post-silking N uptake, with the remaining obtained from remobilization of vegetative N [4]. Compared with older senescent hybrids, stay-green cultivars typically show higher post-silking N uptake and lower N remobilization [9–14]. Grain N concentration has been gradually decreasing with the use of modern stay-green cultivars [15, 16], suggesting that decreased N remobilization may have a negative effect on grain N concentration. Increasing N remobilization efficiency might be an effective strategy to increase grain N concentration and N utilization efficiency [14].

During grain-filling in maize, N uptake and N remobilization are not independent processes. Absorbed N is not transported directly to grain; instead, it is first carried into vegetative organs and then at a later stage translocated into grain. In vegetative organs, N input and export occur simultaneously [6, 17, 18]. To trace these complex nitrogen trajectories, the stable N isotope ^15^N has become an essential technique. By using this method, scientists found that in maize the recovery of fertilizer N ingrain and silage was about 50% [19, 20]. In field experiments, the stalks and leaves are the major source of kernel N, with the roots having a much smaller contribution [17, 21–24]. Nevertheless, information is scare concerning the dynamic distribution of pre- and post-silking N in each organ, information that is essential for understanding their relative contribution to grain N accumulation.

The regulation of pre-silking N remobilization and post-silking N uptake in relation to grain N accumulation is poorly understood. In one study, the average proportion of post-silking N allocated to the kernels was 83% [7], but in general the remobilization of pre-silking N and post-silking N uptake are expected to be affected by genotype as well as external environmental factors including N supply itself [25, 26] and within-plant source-sink relationship [21, 27–29]. In addition, Rajcan and Tollenaar [28] concluded that the proportion of N derived from post-silking N uptake in grain is positively associated with the competition among sinks. The accumulation of starch and protein in grain seems to be closely linked [30]. Reed *et al*. [31] suggested that the supply of post-silking N to grain may be limited by the amount of photosynthate partitioned for nitrate uptake and reduction during grain filling. Nevertheless, how the grain sink coordinates post-silking N uptake and the remobilization of pre-silking N is largely unknown.

Most of the studies on source-sink regulation on pre-silking N remobilization and post-silking N uptake are based on the apparent balance of the N in different organs. However, the export of pre-silking N and the import of post-silking N happen simultaneously in the vegetative organs. The impact of grain sink on pre-silking N remobilization and post-silking N uptake cannot be evaluated accurately without the use of ^15^N labeling. Here, we used ^15^N to analyze the dynamic behavior of both pre- and post-silking N in various organs of maize throughout grain filling in a 2-year greenhouse experiment. Source and sink adjustment (prevention of pollination, removal of grains or partial pollination, defoliation, and covering leaves with aluminum foil) was used to elucidate the effect of source-sink relationships on pre-silking N remobilization and post-silking N uptake. This information is critical to identify the key physiological processes regulating pre-silking N remobilization or post-silking N uptake and grain N accumulation.

## Material and Methods

### Plant culture

Experiments were conducted in 2013 and 2014 in a greenhouse at China Agricultural University, Beijing, China. A stay-green hybrid of maize (*Zea mays* L.), cv. ZD958, was used. Maize plants were grown in pots (32.5 cm wide × 26 cm high) for 126 days in 2013 and 128 days in 2014. Each pot contained 14 kg clayey soil. The chemical characteristics of the soil in 2013 and 2014, respectively, were as follows: pH, 8.27 and 8.30; total N, 0.50 and 0.63 g kg^−1^; Olsen-P, 2.75 and 5.86 mg kg^−1^; NH_4_Ac-K, 103.9 and 94.3 mg kg^−1^; organic matter, 8.3 and 10.5 g kg^−1^. Before planting, 4.3 g urea (46% N), 16.9 g calcium superphosphate (16% P_2_O_5_) and 7.7 g potassium sulfate (52% K_2_O) were mixed into the soil of each pot.

Two maize seeds per pot were planted on 18 April 2013 and 13 April 2014. Plants were thinned to one seedling per pot 12 days after germination and were harvested at physiological maturity (when black-layer formed in the grains) on 23 August 2013 and 17 August 2014. All the pots were arranged in a randomized design, and were re-randomized every two weeks. Plants were watered regularly to avoid water stress. At silking stage, 2L of Hoagland’s solution was added to each pot.

### ^15^N labeling

Two ^15^N labeling treatments were applied, one at the V6 stage (when the sixth leaf is expanded) and the other at silking. A solution of 2.5 mg ^15^N-labeled potassium nitrate (at 10% ^15^N atom excess) in 1 L water was applied to soil surface of each pot. The same amount of potassium nitrate was applied to the control pots. Gallais *et al*. [7] reported that almost all of the ^15^N applied at V6 stage is taken up and assimilated by plants before silking. Consistent with their results, we observed that when applied at the V6 stage, ^15^N did not increase in plants after silking. To check for differences of plants in ^14^N and ^15^N absorption, we additionally set up a control without ^15^N labeling in 2013. No differences in ^14^N and ^15^N absorption were observed.

### Source-sink adjustments

Five source-sink adjustment treatments were applied to plants labeled with ^15^N at V6 stage: (i) control; (ii) no pollination; (iii) reduced sink; (iv) defoliation; and (v) covering of middle leaves. In the control treatment, the plants were fully pollinated. For the no-pollination treatment, the tassels and silks upon appearance were covered with paper bags to prevent pollination. As a result, no grains were formed on the cob. For the reduced-sink treatment, about half of the grains were removed using a knife at 30 days after silking (DAS) in 2013. Considering that N remobilization at this stage may be largely finished, this treatment was adjusted in 2014 in the following way. At silking stage, around one-third of the silks were covered with paper bags, and the remaining two-thirds were pollinated manually. For the defoliation treatment, two leaves above and two leaves below the ear-leaf were removed at the silking stage. Considering that defoliation treatment changes N content in total vegetative organs, a leaf shading treatment was added in 2014. For this treatment, two leaves above and two leaves below the ear-leaf were covered with aluminum foil to prevent photosynthesis from silking until physiological maturity. This treatment was only conducted in 2014. Each treatment had four replicates.

### Plant sampling

Plants were sampled at silking (on 2 July 2013 and 28 June 2014) and every 10 days after silking (DAS) until physiological maturity. The sampled plants were divided into roots, stems (including sheaths and tassels), leaves, cobs, husks, and grains. All senesced leaves were carefully collected. After sampling, all plant organs were heat-treated at 105°C for 30 min, dried at 70°C to a constant weight, weighed to obtain dry matter weight (DM), and then ground into fine powder for N measurement. Four uniform plants were collected per treatment on each sampling date.

### Determination of N content and ^15^N abundance

Subsamples (40 to 80 mg) of the ground samples were carefully weighed in aluminum capsules and used for total N determination in a Flash 2000 Organic Elemental Analyzer (Thermo Fisher Scientific, Villebon, France). The abundance of ^15^N was analyzed by isotope ratio mass spectrometry (DELTA Plus XP; ThermoFinnigan, Germany).

### ^15^N calculations

Abundance of ^15^N, defined for a given organ as the ratio of the amount of ^15^N to the total amount of N (^14^N + ^15^N), was calculated according to Gallais et al. [7] for the ^15^N labeling treatment at the V6 stage. Q_silk(o)_ and Q_mat(o)_, which represent plant organ ^15^N amount at respectively, silking and maturity, as well as P_silk(o)_ and P_mat(o)_, which represent the proportion of ^15^N in each organ at silking and maturity, as follows:

$$Q_{silk}{}_{\left(o\right)}=N_{silk}{}_{\left(o\right)}*(a_{silk}{}_{\left(o\right)}-a_{0})$$ (1)

$$Q_{mat}{}_{\left(o\right)}=N_{mat(o)}*(a_{mat}{}_{\left(o\right)}-a_{0})$$ (2)

$$P_{silk(o)}=Q_{silk}{}_{\left(o\right)}/Q_{silk(wp)}$$ (3)

$$P_{mat(o)}=Q_{mat}{}_{\left(o\right)}/Q_{mat(wp)}$$ (4)

where a_0_ is the natural ^15^N abundance, a_silk(o)_ and a_mat(o)_ are plant organ ^15^N abundances at silking and maturity, N_silk(o)_ and N_mat(o)_ are the amounts of total N in the plant organ at silking and maturity, and Q_mat(wp)_ and Q_silk(wp)_ are the total summarized ^15^N abundances in the whole plant during these time.

The quantity of N coming from post-silking absorption (N_abs_) and from remobilization of the N accumulated before silking (N_rem_) were calculated for the whole plants using the following equation:

$$N_{abs(wp)}=N_{mat(wp)}-N_{silk(wp)}$$ (5)

Because ^15^N applied at V6 was no longer absorbed by plants after silking, N remobilization—the process of ^15^N redistribution to different organs—was calculated for each organ as follows:

$$N_{rem(o)}=N_{silk(wp)}*(P_{silk(o)}-P_{mat(o)})$$ (6)

$$N_{abs(o)}=N_{mat(o)}-(N_{silk(o)}-N_{rem(o)})$$ (7)

$$N_{con}\%=N_{rem/abs}/N_{grain}*100$$ (8)

$$N_{res(o)}=N_{silk(o)}-N_{rem(o)}$$ (9)

where N_abs(wp)_ is whole-plant N after silking, N_mat(wp)_ and N_silk(wp)_ are total N content in whole-plant at silking and maturity, respectively, N_rem(o)_ is the quantity of remobilized N in each organ, N_abs(o)_ is the quantity of absorbed N in each organ, N_res(o)_ is the quantity of pre-silking N residual in each organ, P'_mat(o)_ is the proportion of ^15^N label applied at silking in each mature organ and N_con_% is the contribution of remobilized or absorbed N (N_rem/abs_) to grain N (N_grain_). The same formulas were applied to calculate N remobilization at other sampling stages.

Analysis of variance (ANOVA) was conducted using the GLM procedure of IBM SPSS Statistics 20.0. Significant differences among means were separated by LSD at the *P*≤ 0.05 probability level. Plant DM and N uptake and remobilization were subjected to two-way ANOVA to assess the effects of source-sink treatments. Figures were constructed in GraphPad Prism 6.0, and regression analyses were conducted in Curve Expert 1.3.

## Results

### Plant growth and N accumulation

At silking stage, the whole plant DM was significantly lower in 2013 than in 2014 (Table 1), possibly because hours of sunshine were less in 2013 than in 2014 (S1 Fig). At physiological maturity, no difference was observed in grain yield between two years but the DM of total vegetative organs in 2014 was 22% higher than in 2013. In both years, post-silking DM production was ~12% greater than grain yield. This surplus DM mainly accumulated in cobs, leaves and husks (S1 Table). Whole plant N accumulation at silking and maturity was 8% higher in 2013 than in 2014 (Table 1). At maturity, the higher N content in 2014 was in the grains, while N amount in total vegetative organs was the same between the two years. At maturity, N concentrations in roots, stems, husks and cobs, but not leaves, were higher in 2013 than in 2014 (S2 Table).

**Table 1:** Dry matter (DM) and nitrogen (N) accumulation in vegetative organs in 2013 and 2014.

		Dry matter (g plant^-1^)				Nitrogen (g plant^-1^)			
		Root	Shoot	Total vegetative organs	Grains	Root	Shoot	Total vegetative organs	Grains
At silking	2013	10.6a	57.9b	68.5b		0.20a	1.23a	1.43a	
	2014	11.7a	73.1a	84.8a		0.13b	1.21a	1.33b	
At maturity	2013	9.1a	68.6b	77.7b	78.0a	0.16a	0.73a	0.89a	1.45a
	2014	9.1a	85.5a	94.7a	76.7a	0.13a	0.70a	0.82a	1.29b

Values followed by different letters indicate significant differences (P < 0.05) in the same organs between two years.

### Distributions of pre- and post-silking ^15^N in organs and their contributions to grain N

**Fig 1:**
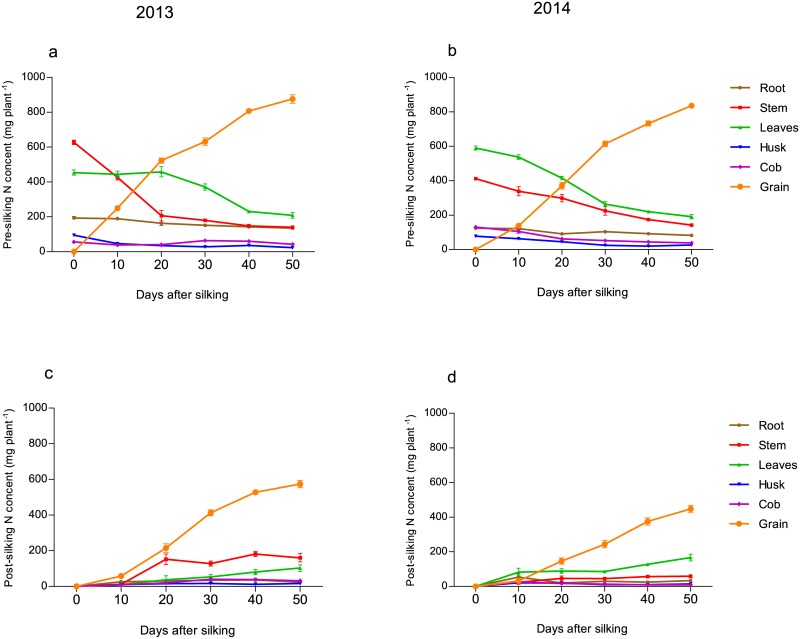
Changes in pre-silking (a and b) and post-silking (c and d) ^15^N content of different maize organs from silking to physiological maturity in 2013 and 2014. All data are based on ^15^N results. Data are means ± SE.

To understand the dynamic allocation of pre-silking N in different organs during grain-filling, plants were labeled with ^15^N at the V6 stage. The amount of pre-silking ^15^N in grains began increasing at the onset of grain-filling, indicating that pre-silking N was readily remobilized from vegetative organs into grain (Fig 1a and 1b). In 2013, the remobilization of pre-silking ^15^N among vegetative organs started much earlier from the stem (right after silking) than from leaves (20 days after silking). While in 2014, the remobilization of pre-silking ^15^N occurred at the start of silking in both leaves and stems. The amount of pre-silking^15^N remained relatively constant in roots throughout grain-filling.

We also applied ^15^N labeling at silking to understand the dynamic allocation of N absorbed subsequently (Fig 1c and 1d). In agreement with the results described above (Table 1), plants took up much more ^15^N during the post-silking phase in 2013 than in 2014. Between the two years, there was significant difference in the accumulation of post-silking ^15^N in grain. In vegetative organs, the accumulation of post-silking ^15^N in the leaves took place until physiological maturity.

**Fig 2:**
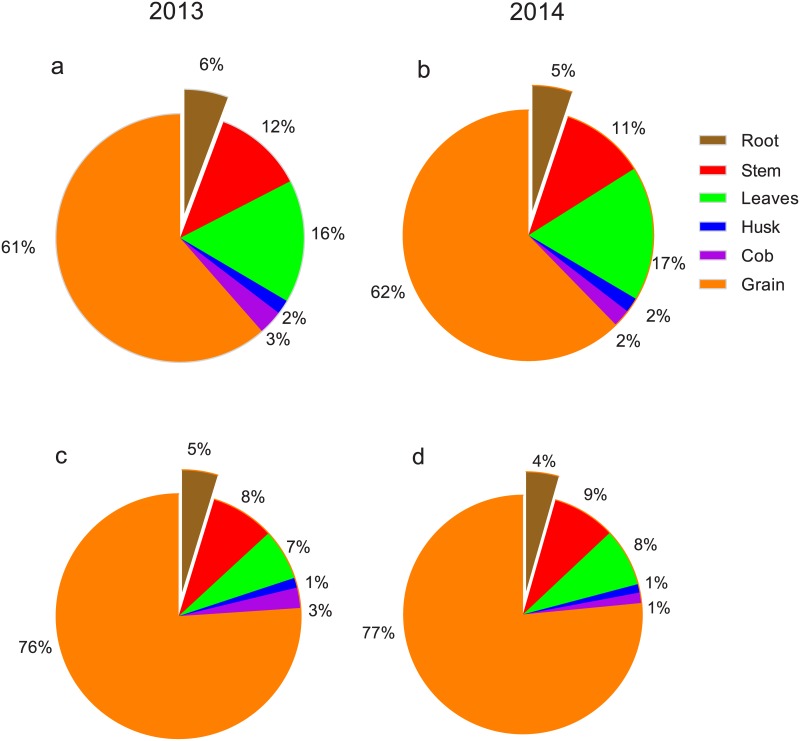
Percentages of pre-silking (a and b) and post-silking^15^N (c and d) distributed in different organs at physiological maturity. Pre- and post-silking^15^N labeling was applied at V6 stage and silking stages, respectively.

Although a large difference was observed in pre- and post-silking N uptake pattern between the 2 years, the distribution of pre- and post-silking ^15^N in each organ at physiological maturity differed only slightly. In regard to the distribution of total pre-silking N, ~60% was mobilized to grains, while around 10 to 20% remained in stems and leaves, and 5% or less remained in husks, cobs, and roots (Fig 2a and 2b). As for the allocation total post-silking nitrogen, the grains were even more dominant, receiving more than three quarters of the total, with less than 10% going to stems and leaves (Fig 2c and 2d).

**Fig 3:**
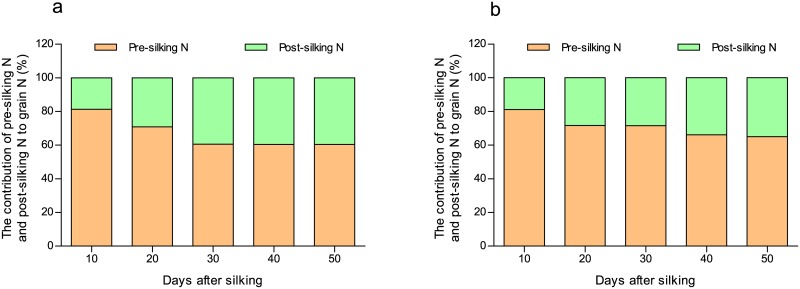
Changes in the percentage contribution of pre- and post-silking nitrogen (N) to grain N in 2013 (a) and 2014 (b). Data are based on the results of ^15^N labeling at the V6 stage.

Using the data for pre-silking ^15^N accumulation in grain, we estimated the dynamic contribution of pre- and post-silking N to grain (Fig 3). In both years, the remobilized pre-silking N had a greater contribution to grain N throughout grain-filling, though the contribution of post-silking N uptake gradually increased with the grain filling process. As assessed by the ^15^N method, the contribution of remobilized pre-silking N to grain N was 60% in 2013 and 65% in 2014.

### Effects of sink regulation on post-silking N uptake and remobilization of pre-silking N

**Fig 4:**
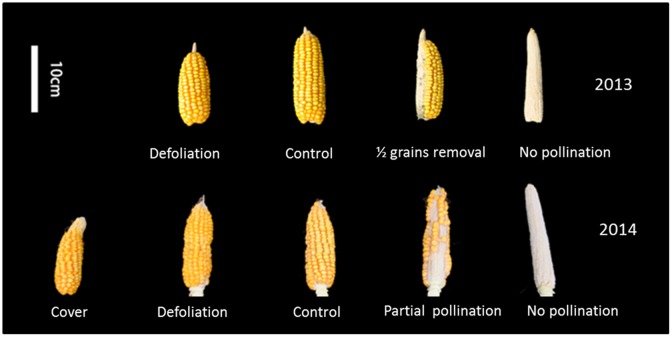
Effects of source and sink regulation on grain development. Other than a 50% grain removal treatment performed 30 days after silking in 2013, all treatments were conducted at the silking stage.

**Fig 5:**
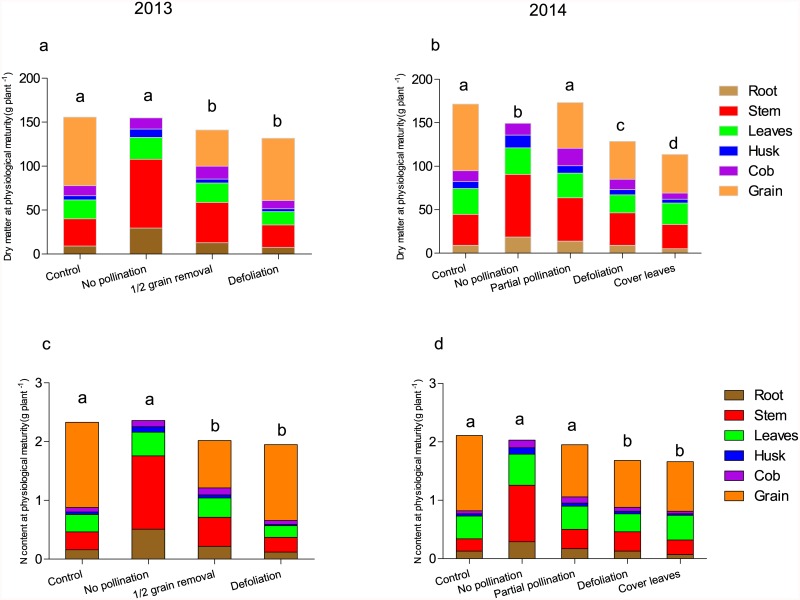
Effect of source and sink regulation on dry matter mass (a and b) and nitrogen (N) content (c and d) in different organs at physiological maturity. Data are means of four replicates. Different letters indicate significant differences in whole plant dry matter or N content between different treatments (*P*< 0.05).

When the grain sink was removed by preventing pollination, the whole-plant DM was reduced (Table 2, Figs 4, 5a and 5b). However, the vegetative organ DM increased and the total plant N accumulation was hardly affected. Compared to that of the control plants, DM and N content in the roots and stems of the sink-removed plants were much higher (Fig 5). The results of the ^15^N-tracing experiment suggest that post-silking N uptake increased in all vegetative organs as a consequence of grain sink removal (Table 3). Nevertheless, large remobilization of pre-silking N still took place in the leaves (Table 3). In the non-pollinated plants compared to control plants, residual pre-silking nitrogen accumulated to a greater extent in the stems, roots, husks, and cobs, but was equal in leaves.

**Table 2:** Effects of source and sink regulation on grain yield and its components, grain nitrogen concentration, plant dry matter, and harvest index.

Year	Treatments	Yield (g plant^−1^)	Grain number (ear^−1^)	100 grain weight (g)	Grain N concentration (mg g^−1^)	Plant N (g plant^-1^)	Plant DM (g plant^−1^)
2013	Control	78.0±1.5a	296±14a	26.4±1.2a	18.6±0.3b	2.34±0.05a	155.7±1.5a
	No pollination					2.38±0.04a	154.9±4.4a
	1/2 grain removal	41.1±0.7c	154±3b	26.8±0.4a	19.6±0.4a	2.01±0.06b	141.0±3.4b
	Defoliation	70.9±2.5b	268±7a	25.9±0.9a	18.1±0.2b	1.93±0.05b	131.8±5.1b
2014	Control	76.7±0.8a	320±6a	24.0±0.6ab	16.8±0.1b	2.10±0.04a	171.4±0.6a
	No pollination					2.03±0.05a	149.4±4.1b
	Partial pollination	52.4±1.6b	213±12b	24.8±1.2a	17.1±0.5b	1.95±0.03a	173.2±5.8a
	Defoliation	43.4±1.6c	181±9c	24.1±1.3ab	18.5±0.1a	1.68±0.08b	128.6±6.7c
	Cover leaves	44.7±2.6c	223±11b	20.3±2.0b	19.0±0.7a	1.65±0.06b	113.7±5.1d

Data are means ± SE (n = 4). Within columns, different letters indicate significant differences at P < 0.05 between different source sink treatments in a year.

**Table 3:** Effects of sink regulation on post-silking N uptake, remobilization of pre-silking accumulated N and residual pre-silking N in each organ at physiological maturity. All data are based on ^15^N results.

	Year	Treatments	Organs						
			Root	Stem	Leaves	Husk	Cob	Total vegetative organs	Grain
Post-silking N uptake (mg plant^-1^)	2013	Control	24.7b	160.5b	102.8a	16.4b	32.1a	336.4b	573.8a
		No pollination	213.5a	544.1a	130.3a	35.0a	33.1a	955.9a	
		1/2 grain removal	44.4b	193.6b	23.8b	16.3b	41.3a	319.3b	265.5b
	2014	Control	38.5b	33.1b	239.5a	4.8b	4.3b	320.1b	447.7a
		No pollination	122.6a	315.1a	185.2b	36.4a	31a	690.2a	
		Partial pollination	55.8b	34.6b	140.8c	9.4b	23.3a	263.8b	349.6b
Remobilization of pre-silking N (mg plant^-1^)	2013	Control	57.9a	487.8a	245.0a	72.4a	13a	876.1a	
		No pollination	-102.6c	-77.2c	157.6b	41.4b	-19.2a	0	
		1/2 grain removal	15.5b	331.0b	150.9b	67.9a	-27.6a	537.7b	
	2014	Control	39.3a	231a	441.4a	41.1a	83.4a	836.2a	
		No pollination	-43.8c	-245c	249.5c	2.7b	36.5b	0	
		Partial pollination	7.8b	121.7b	336.2b	33.9a	45.1b	544.7b	
Residual pre-silking N (mg plant^-1^)	2013	Control	135.4c	139.5c	209.0b	22.4b	42.8b	549.2c	
		No pollination	295.9a	704.5a	296.4a	53.5a	75.1a	1425.3a	
		1/2 grain removal	177.7b	296.3b	303.1a	27.0b	83.5a	887.6b	
	2014	Control	85.1c	180.8c	148.4c	36.7b	47.4b	498.4c	
		No pollination	168.1a	656.8a	340.2a	75.1a	94.3a	1334.5a	
		Partial pollination	116.7b	290.1b	253.6b	43.9b	85.7a	789.9b	

Data are means ± SE (n = 4). Within columns, different letters indicate significant differences at P < 0.05 between different source: sink treatments in the same organ in a year. Negative values indicate there was no pre-silking N remobilized from the organ.

In 2013, the removal of half of the grains per ear at 30 DAS resulted in the reduction of grain yield per plant by one-half. However, DM of the roots and the stem increased by 40% and 48% respectively. In accord with this, compared to the control, the root and stem N content increased by 40% and 63% respectively, and grain N was reduced by 45% (Table 2, Figs 4, 5a and 5c). According to the results of the ^15^N-tracing experiment, post-silking N accumulation was significantly reduced in the grain and leaves (Table 3). The remobilization of pre-silking N was reduced in the roots, stems and leaves, but a net accumulation of pre-silking N occurred in cobs. In comparison to the control, a larger amount of residual pre-silking N was present in the roots, stems, and leaves of plant subjected to the grain removal treatment.

In 2014, when approximately one-third of silks were covered to prevent pollination, grain yield was reduced by 32% (Table 2, Fig 4). Because of compensatory growth of the vegetative organs (Fig 5b and 5d), the whole-plant DM and N accumulation were unchanged (Table 2). The ^15^N-tracing experiment indicated a slight increase in post-silking N in the cobs, but a decrease in grain (Table 3). The total remobilization of pre-silking N was reduced by 35%. This reduction occurred to a greater extent in the stem, leaves and roots.

### Effects of source regulation on post-silking N uptake and remobilization of pre-silking N

When source size was reduced by removing four leaves at silking, grain yield was reduced by 9% in 2013 but by 43%in 2014, respectively (Table 2). Despite the difference in yield between years, whole-plant dry matter and nitrogen content decreased by about 20% in both years (Table 2). According to the results of the ^15^N tracing experiment, the accumulation of post-silking N in 2013 and 2014 was reduced by 48% and 12% in the vegetative organs and by 19% and 33% in the grains, respectively (Table 4). In 2013, when the yield reduction from defoliation was only 9%, no difference was found in the total amount of remobilized pre-silking N in vegetative organs (Table 4). In 2014 when grain yield was reduced by 43%, defoliation significantly reduced the remobilization of pre-silking N because less N was remobilized from the remaining leaves and stems (Table 4). As a result, more residual pre-silking N was present in the remaining leaves and stems of plants subjected to the defoliation treatment in 2014 (Table 4).

**Table 4:** Effects of source regulation on post-silking N uptake, remobilization of pre-silking accumulated N and residual pre-silking N in each organ at physiological maturity. All the data are based on ^15^N results.

	Year	Treatments	Organs							
			Root	Stem	Treated leaves	Remaining leaves	Husk	Cob	Total vegetative organs	Grain
Post-silking N uptake (mg plant^-1^)	2013	Control	24.7a	160.5a	56.6	46.2a	16.4a	32.1a	336.4a	573.8a
		Defoliation	1.3b	95.4b		45.6a	11.6a	20.8b	174.7b	464.1b
	2014	Control	38.5a	33.1ab	72.7a	166.8a	4.8a	4.3ab	320.1a	447.7a
		Defoliation	48.5a	66.1a		147.5ab	5.2a	13.8a	281.0ab	300.5b
		Cover leaves	7.7b	0.9b	10.0b	79.3b	3.1a	1.8b	102.7b	232.7b
Remobilization of pre-silking N (mg plant^-1^)	2013	Control	57.9a	487.8a	71.9	173.1a	72.4a	13.0a	876.1a	
		Defoliation	69.8a	480.9a		177.9a	84.2a	9.2a	821.9a	
	2014	Control	39.3a	231.0a	140.6a	300.8a	41.1a	83.4ab	836.2a	
		Defoliation	43.0a	144.6b		201.3b	45.3a	68.8b	502.8c	
		Cover leaves	64.1a	152.5b	94.9b	169.8b	46.1a	88.3a	615.7b	
Residual pre-silking N (mg plant^-1^)	2013	Control	135.4a	139.5a	67.0	142.0a	22.4a	42.8a	549.2a	
		Defoliation	123.4a	146.5a		137.2a	10.7b	46.7a	464.4b	
	2014	Control	85.1a	180.8b	87.4b	61.0b	36.7a	47.4ab	498.4c	
		Defoliation	81.5a	267.3a		160.5a	32.6a	62.0a	603.8b	
		Cover leaves	60.3a	259.3a	133.1a	192.0a	31.8a	42.6b	708.9a	

Data are means ± SE (n = 4). Within columns, different letters indicate significant differences at P < 0.05 between different source: sink treatments in the same organ in a year.

When four leaves were covered with aluminum foil at silking in 2014, grain number per ear, grain yield, and whole-plant dry mass decreased by about one third (Table 2, Figs 4 and 5b), as did nitrogen content in grain and in the whole plant (Fig 5d). According to the ^15^N tracing results, post-silking N uptake as a result of the leaf-covering treatment was greatly reduced in both vegetative organs and grains (Table 4). Remobilization of pre-silking N was reduced in the remaining leaves, stem and the treated leaves, but increased in the roots.

## Discussion

### Behaviors of pre- and post-silking N and their contributions to grain N

The processes of pre-silking N export and post-silking N import occur simultaneously in stems and leaves, suggesting that proteins turnover in these organs [32]. However, the export of remobilized N was greater than the import of post-silking N, which resulted in a net reduction in N concentration and N content in aboveground vegetative organs (S2 and S3 Tables). In grain, N importation from both remobilized N and post-silking N occurred synchronously. However, at early in grain filling, remobilized N had the greatest contribution to grain N; later on, the contribution of post-silking N gradually increased (Fig 3). Masclaux-Daubresse *et al*. [8] also suggested that pre-silking N is preferentially used for grain development.

Here, the final distribution of pre- and post-silking N in each vegetative organ at physiological maturity was quite similar between the 2 years. On average, the leaves contained 17% of the total pre-silking ^15^N and 7% of the total post-silking ^15^N, while stems contained 11% of the total pre-silking ^15^N and 9% of the total post-silking ^15^N. As the major N sinks, grains accumulated 62% of the pre-silking ^15^N and 76% of the post-silking ^15^N (Fig 2). Cliquet *et al*. [17] reported that 42% of labeled ^15^N at the stem elongation stage is recovered in grains. Weiland and Ta [23] estimated that under high and low N fertility, 60% and 62% respectively of labeled ^15^N at the 12-leaf stage is allocated to grains at physiological maturity. In addition, Gallais *et al*. [7] reported that 83% of post-silking N is allocated to kernels, with the remaining 17% allocated to the stover. Taken together, these data imply that grain N content is more likely to increase when post-silking N uptake is high. Consistent with this assumption, higher N uptake during the post-silking stage in 2013 brought about a higher grain N concentration (S2 Table), similar to results seen in other hybrids [10].

### Regulation of post-silking N accumulation

Previous studies using various varieties [14, 33, 34] and N levels [35] show that higher post-silking DM accumulation is more likely to lead to higher post-silking N uptake and grain yield. Under normal growth conditions, almost all photosynthate is used for grain starch and protein synthesis, which are processes requiring N [30]. With increasing DM production, more carbon can flow into the roots to enhance N uptake [36]. The results from source-sink adjustment treatments support the idea that aboveground DM accumulation regulates post-silking N uptake. Covering four leaves with aluminum foil or removing them is expected to reduce total canopy photosynthesis. When these treatments were applied, the DM weight of all organs was reduced by 15 to 34% (Table 2). Accordingly, post-silking N accumulation was reduced by 24% to 56% (Table 4).

When the grain sink was completely removed by preventing pollination in both years, post-silking N accumulation was hardly affected (Table 3). Apparently, the reason is that the growth of stems, husks, and roots was greatly enhanced, which became alternative sinks for N deposition. Similar results were found for grain removal in 2013 and partial pollination in 2014 (Fig 5), although to different extents. Pan *et al*. [37] suggested that grain filling and root functions compete for photosynthate, with grain filling usually having a higher priority than root functions. When the grain sink is eliminated, more photosynthate is allocated to the roots and stem. The enhancement of root growth might in turn help promote post-silking N uptake [38].

### Regulation of pre-silking N remobilization

Although N accumulation in grain was limited during the first 10 days after silking, N remobilization from the leaves and stem was started, together with post-silking N uptake. Therefore, the onset of N remobilization from the leaves and stem seemed not triggered by the balance between grain N demand and N supply from N uptake. Consequently, the current data counter the idea that the occurrence of N remobilization is due to the supply of post-silking N uptake being insufficient to meet grain development demands [8, 39]. Ta and Weiland [40] also concluded that N remobilization is independent of post-silking N uptake.

**Fig 6:**
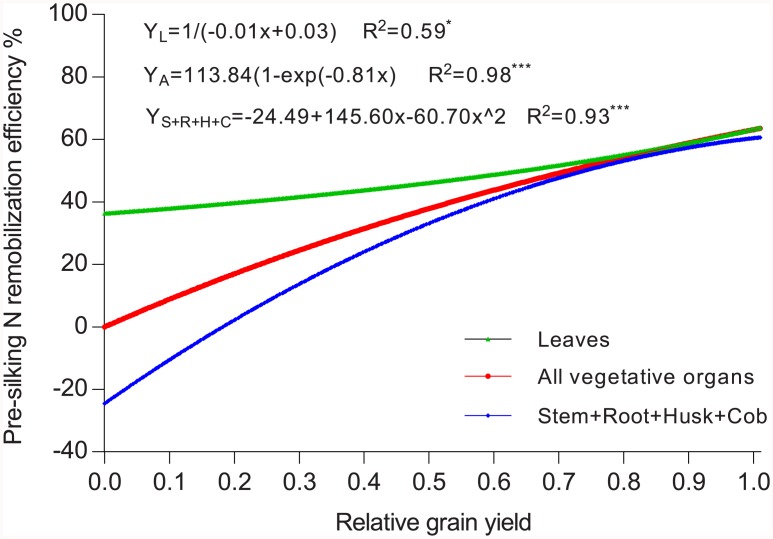
Correlations between relative grain yield and pre-silking nitrogen remobilization efficiency of the total vegetative organs, the leaves and the remaining vegetative organs (stem + root + husk + cob). Data from all source and sink treatments during 2 years were pooled for the correlation analysis. The grain yield in the control treatment in each year was set to 100%. *P < 0.05; *** *P* < 0.001.

To understand the relationship between grain yield (sink size) and pre-silking N remobilization efficiency in vegetative organs, we normalized all ^15^N experimental data by setting the highest value of grain yield each year to 100% (Fig 6). For leaves, pre-silking N remobilization efficiency was 33% even though grain yield was zero (no pollination), suggesting that the start of N remobilization from the leaves is independent of the existence of a grain sink. Without a grain sink, this remobilized N from the leaves may be accumulated in other organs (stems, roots, and husk + cob). In a classic experiment, Christensen *et al*. [21] also found that N export from leaves continues even when ears were removed.

It is seen in Fig 6 that, when there is a grain sink, the remobilization efficiency of pre-silking N depends largely on grain yield, thus grain N requirement. By simulation, the maximum N remobilization efficiency in the present study was similar for the leaves (63%) and the remaining vegetative organs (60%). In 2013, when the yield reduction from defoliation was only 9%, the remobilization of pre-silking N was hardly affected (Table 4). But in 2014, when grain yield was reduced by 43% by defoliation, total remobilization of pre-silking N was reduced significantly. Similarly, compared to the control, lower grain yield in the leaf-covering treatment also resulted in a significant decreased in N remobilization (Table 4). Grain N requirements thus appear to be fulfilled by regulating both pre-silking N remobilization and post-silking N uptake.

## Conclusion

Nitrogen export from the leaves could start right after the onset of pollination when there was no symptom of leaf senescence. Accordingly, grain N was mostly contributed by pre-silking N at early rather than late grain filling stage. Compared to pre-silking N, a larger fraction of post-silking N was allocated to grain, implying that grain N content is more likely to increase when more post-silking N uptake occurs. The amount of post-silking N uptake was largely regulated by post-silking DM accumulation in both grains and vegetative organs. The remobilization efficiency of the total pre-silking N was regulated by grain N demand, however, N remobilization from the leaves was not dependent on the existence of the grains. It seems that the leaf *per se* is not the factor determining the efficiency of N remobilization in the leaves.

## Supporting Information

S1 Fig Hours of accumulated sunshine and temperature during maize development in 2013 and 2014. (TIF)

S1 Table Dry matter accumulation and partitioning within the plant from silking to physiological maturity. (DOC)

S2 Table Dynamic changes of N concentration in each organ from silking to physiological maturity. (DOC)

S3 Table Dynamic changes of N content in different organs from silking to physiological maturity. (DOC)

## References

[1] Raun WR, Johnson GV. Improving nitrogen use efficiency for cereal production. *Agron J*. 1999; 91: 357–363.

[2] Ju XT, Xing GX, Chen XP, Zhang SL, Zhang LJ, Liu XJ, et al Reducing environmental risk by improving N management in intensive Chinese agricultural systems. *P Natl AcadSciUsa*. 2009; 106: 3041–3046. 10.1073/pnas.0813417106 PMC2644255 19223587

[3] Mueller ND, Gerber JS, Johnston M, Ray DK, Ramankutty N, Foley JA. Closing yield gaps through nutrient and water management. *Nature*. 2012; 490: 254–257. 10.1038/nature11420 22932270

[4] Hirel B, Le Gouis J, Ney B, Gallais A. The challenge of improving nitrogen use efficiency in crop plants: towards a more central role for genetic variability and quantitative genetics within integrated approaches. *J Exp Bot*. 2007; 58: 2369–2387. 17556767 10.1093/jxb/erm097

[5] Ciampitti IA, Vyn TJ. Grain nitrogen source changes over time in maize: areview. *Crop Sci*. 2013; 53: 366–377.

[6] Gallais A, Coque M, Quillere I, Prioul J, Hirel B. Modelling post-silking nitrogen fluxes in maize (*Zea mays* L) using N-15-labelling field experiments. *New Phytol*. 2006; 172: 696–707. 17096795 10.1111/j.1469-8137.2006.01890.x

[7] Gallais A, Coque M, Le Gouis J, Prioul JL, Hirel B, Quillere I. Estimating the proportion of nitrogen remobilization and of post-silking nitrogen uptake allocated to maize kernels by nitrogen-15 labeling. *Crop Sci*. 2007; 47: 685–693.

[8] Masclaux-Daubresse C, Reisdorf-Cren M, Orsel M. Leaf nitrogen remobilisation for plant development and grain filling. *Plant Biology*. 2008; 101: 23–36. 10.1111/j.1438-8677.2008.00097.x 18721309

[9] Ma BL, Dwyer LM. Nitrogen uptake and use of two contrasting maize hybrids differing in leaf senescence. *Plant Soil*.1998; 199: 283–291.

[10] Mi GH, Liu JA, Chen FJ, Zhang FS, Cui ZL, Liu XS. Nitrogen uptake and remobilization in maize hybrids differing in leaf senescence. *J Plant Nutr*.2003; 26: 237–247.

[11] Valentinuz OR, Tollenaar M. Vertical profile of leaf senescence during the grain-filling period in older and newer maize hybrids. *Crop Sci*. 2004; 44: 827–834.

[12] Subedi KD, Ma BL. Nitrogen uptake and partitioning in stay-green and leafy maize hybrids. *Crop Sci*. 2005; 45: 740–747.

[13] Pommel B, Gallais A, Coque M, Quillere I, Hirel B, Prioul JL, et al Carbon and nitrogen allocation and grain filling in three maize hybrids differing in leaf senescence. *Eur J Agron*. 2006; 24: 203–211.

[14] Chen YL, Xiao CX, Chen XC, Li Q, Zhang J, Chen FJ, et al. Characterization of the plant traits contributed to high grain yield and high grain nitrogen concentration in maize. *Field Crop Res*. 2014; 159: 1–9.

[15] Duvick DN, Cassman KG. Post-green revolution trends in yield potential of temperate maize in the north-central United States. *Crop Sci*. 1999; 39: 1622–1630.

[16] Chen X, Chen F, Chen Y, Gao Q, Yang X, Yuan L, et al Modern maize hybrids in Northeast China exhibit increased yield potential and resource use efficiency despite adverse climate change. *Global Change Biol*. 2013; 19: 923–936. 10.1111/gcb.12093 23504848

[17] Cliquet JB, Deleens E, Mariotti A. C and N mobilization from stalk and leaves during kernel filling by C-13 and N-15 tracing in *Zea Mays* L. *Plant Physiol*. 1990; 94: 1547–1553. 16667888 10.1104/pp.94.4.1547 PMC1077419

[18] Pan WL, Camberato JJ, Moll RH, Kamprath EJ, Jackson WA. Altering source-sink relationships in prolific maize hybrids-consequences for nitrogen uptake and remobilization. *Crop Sci*. 1995;35: 836–845.

[19] Cliquet JB, Deleens E, Bousser A, Martin M, Lescure JC, Prioul JL, et al Estimation of carbon and nitrogen allocation during stalk elongation by 13-C and 15-N tracing in *Zea Mays* L. *Plant Physiol*. 1990; 92: 79–87. 16667269 10.1104/pp.92.1.79 PMC1062251

[20] Tran TS, Giroux M. Fate of 15N-labelled fertilizer applied to corn grown on different soil types. *Can J Soil Sci*. 1998; 78: 597–605.

[21] Christensen L, Below F, Hageman R. The effects of ear removal on senescence and metabolism of maize. *Plant Physiol*. 1981; 68, 1180–1185. 16662071 10.1104/pp.68.5.1180 PMC426065

[22] Ta CT. Nitrogen metabolism in the stalk tissue of maize. *Plant Physiol*. 1991; 97: 1375–1380. 16668559 10.1104/pp.97.4.1375 PMC1081174

[23] Weiland RT, Ta TC. Allocation and retranslocation of N-15 by maize (*Zea Mays*. L) hybrids under field conditions of low and high-N fertility. *Aust*. *J*. *Plant Physiol*.1992; 19: 77–88.

[24] Ma BL, Dwyer LM. Stem-infused nitrogen-15 enrichment for evaluation of nitrogen use in maize. *Commun Soil Sci Plan*. 1998; 29: 2459–2470.

[25] Niu J, Peng Y, Li C, Zhang F. Changes in root length at the reproductive stage of maize plants grown in the field and quartz sand. *J Plant Nutr Soil Sc*. 2010; 173: 306–314.

[26] Kant S, Bi YM, Rothstein SJ. Understanding plant response to nitrogen limitation for the improvement of crop nitrogen use efficiency. *J Exp Bot*. 2011; 62: 1499–1509. 10.1093/jxb/erq297 20926552

[27] Crafts-Brandner SJ, Below FE, Harper JE, Hageman RH. Differential senescence of maize hybrids following ear removal: 1. Whole plant. *Plant Physiol*. 1984; 74: 360–367. 16663423 10.1104/pp.74.2.360 PMC1066683

[28] Rajcan I, Tollenaar M. Source: sink ratio and leaf senescence in maize: II. Nitrogen metabolism during grain filling. *Field Crop Res*. 1999; 60: 255–265.

[29] Yan H, Shang A, Peng Y, Yu P, Li C. Covering middle leaves and ears reveals differential regulatory roles of vegetative and reproductive organs in root growth and nitrogen uptake in maize. *Crop Sci*. 2011; 51: 265–272.

[30] Seebauer JR, Singletary GW, Krumpelman PM, Ruffo ML, Below FE. Relationship of source and sink in determining kernel composition of maize. *J Exp Bot*. 2010; 61: 511–519. 10.1093/jxb/erp324 19917600 PMC2803218

[31] Reed AJ, Singletary GW, Schussler JR, Willamson DR, Christy AL. Shading effects on dry matter and nitrogen partitioning, kernel number and yield of maize. *Crop Sci*. 1988; 28: 819–825.

[32] Hirel B, Gallais A. Rubisco synthesis, turnover and degradation: some new thoughts on an old problem. *New Phytol*. 2006; 169: 445–448. 16411947 10.1111/j.1469-8137.2006.01641.x

[33] Antonietta M, Fanello DD, Acciaresi HA, Guiamet JJ. Senescence and yield responses to plant density in stay green and earlier-senescing maize hybrids from Argentina. *Field Crop Res*. 2014; 155: 111–119.

[34] Mu X, Chen F, Wu Q, Chen Q, Wang J, Yuan L, et al Genetic improvement of root growth increases maize yield via enhanced post-silking nitrogen uptake. *Eur J Agron*. 2015; 63: 55–61.

[35] Chen Y, Xiao C, Wu D, Xia T, Chen Q, Chen F, et al Effects of nitrogen application rate on grain yield and grain nitrogen concentration in two maize hybrids with contrasting nitrogen remobilization efficiency. *Eur J Agron*. 2015; 62: 79–89.

[36] Jones DL, Nguyen C, Finlay RD, Carbon flow in the rhizosphere: carbon trading at the soil-root interface. *Plant Soil*. 2009; 321: 5–33.

[37] Pan WL, Camberato JJ, Jackson WA, Moll RH. Utilization of previously accumulated and concurrently absorbed nitrogen during reproductive growth in maize-influence of prolificacy and nitrogen source. *Plant Physiol*. 1986; 82: 247–253. 16665001 10.1104/pp.82.1.247 PMC1056098

[38] Uhart SA, Andrade FH. Nitrogen and carbon accumulation and remobilization during grain filling in maize under different source/sink ratios. *Crop Sci*. 1995; 35: 183–190.

[39] Uhart SA, Andrade FH. Nitrogen deficiency in maize: 1. Effects on crop growth, development, dry matter partitioning and kernel set. *Crop Sci*. 1995; 35: 1376–1383.

[40] Ta CT, Weiland RT. Nitrogen partitioning in maize during ear development. *Crop Sci*. 1992; 32: 443–451.

