# Supplementary material for: Use of the Stable Nitrogen Isotope to Reveal the Source-Sink Regulation of Nitrogen Uptake and Remobilization during Grain Filling Phase in Maize
Source: PLoS One. 2016 Sep 8;11(9):e0162201. doi: 10.1371/journal.pone.0162201 (PMC5015999; doi:10.1371/journal.pone.0162201)
Supplement: S1 Table — (DOC) [file pone.0162201.s002.doc]

**Supporting information:**

Manuscript title:

**Use of the stable nitrogen isotope to reveal the source-sink regulation of nitrogen uptake and remobilization during grain filling phase in maize**

Lan Yang^1^, Song Guo^1, 2^, Qinwu Chen^1^, Fanjun Chen^1^, Lixing Yuan^1^, Guohua Mi^1*^

^1^Center for Resources, Environment and Food Security, College of Resources and Environmental Science, China Agricultural University, Beijing 100193, P.R. China.

^2^ Soil and Fertilizer Research Institute Sichuan Academy of Agricultural Science, Chengdu 610066, P.R. China.

S1 Table. Dry matter accumulation and partitioning within the plant from silking to physiological maturity.

|  | Dry matter accumulation (g plant^-1^) | | | | | | |
| --- | --- | --- | --- | --- | --- | --- | --- |
|  | Root | Stem | Leaves | Husk | Cob | Grain | Whole plant |
| 2013 |  |  |  |  |  |  |  |
| Silking | 10.6±0.4a | 31.2±1.1bc | 19.3±0.6cd | 4.5±0.2a | 2.9±0.1e | | 68.5±2.7e |
| 10DAS | 10.6±0.4a | 29.3±1.4c | 20.6±0.9bc | 5.0±0.3a | 5.7±0.5d | 15.4±0.7e | 86.5±2.8d |
| 20DAS | 10.0±0.6a | 35.5±0.5a | 24.2±0.4a | 5.9±0.2a | 8.9±0.3c | 42.9±0.8d | 127.3±1.3c |
| 30DAS | 9.8±0.5a | 33.8±0.6ab | 22.2±0.5b | 4.5±0.4a | 12.9±0.8a | 53.5±0.8c | 136.5±2.2b |
| 40DAS | 9.2±0.5a | 33.2±1.0abc | 18.4±0.4d | 4.7±0.2a | 14.1±0.5a | 70.9±1.0b | 150.4±1.2a |
| 50DAS | 9.1±0.4a | 31.1±1.2bc | 21.5±0.4b | 4.6±0.5a | 11.5±0.2b | 78.0±1.5a | 155.7±1.5a |
| 2014 |  |  |  |  |  |  |  |
| Silking | 11.6±0.4a | 32.4±0.5c | 24.7±0.7b | 6.8±0.1bc | 9.3±0.2b | | 84.8±1.7f |
| 10DAS | 12.0±0.6a | 38.6±0.8ab | 29.6±0.6a | 9.9±0.2a | 11.6±0.5ab | 8.9±0.8e | 110.6±1.3e |
| 20DAS | 8.5±0.5b | 40.7±1.5a | 29.4±0.7ab | 9.8±1.1a | 12.7±0.7a | 34.3±1.0d | 135.4±1.9d |
| 30DAS | 9.0±0.7b | 34.1±1.2c | 27.3±0.8ab | 6.0±0.8bc | 14.1±0.5a | 54.3±2.1c | 144.8±3.4c |
| 40DAS | 9.2±0.6b | 35.2±0.7bc | 25.3±1.4ab | 5.6±0.8c | 11.9±0.5ab | 68.6±1.1b | 155.7±3.3b |
| 50DAS | 9.1±0.6b | 35.5±1.9c | 29.6±2.8ab | 8.1±0.6ab | 12.3±0.5a | 76.7±1.2a | 171.4±3.8a |
| Source of variance | |  |  |  |  |  |  |
| Year(Y) | NS | *** | *** | *** | *** | *** | *** |
| Sampling dates (S) | *** | *** | *** | *** | *** | *** | *** |
| S*Y | * | ** | NS | * | *** | ** | ** |

Data are means ± SE. Within columns, different letters indicate significant differences at P < 0.05 between different sampling dates in a year. ***, **, * indicate significance at 0.001, 0.01, 0.05 probability level, respectively. NS means not significant at the 0.05 probability level.
