# Supplementary material for: Use of the Stable Nitrogen Isotope to Reveal the Source-Sink Regulation of Nitrogen Uptake and Remobilization during Grain Filling Phase in Maize
Source: PLoS One. 2016 Sep 8;11(9):e0162201. doi: 10.1371/journal.pone.0162201 (PMC5015999; doi:10.1371/journal.pone.0162201)
Supplement: S2 Table — (DOC) [file pone.0162201.s003.doc]

**Supporting information:**

S2 Table. Dynamic changes of N concentration in each organ from silking to physiological maturity.

|  | N concentration (mg g^-1^) | | | | | |
| --- | --- | --- | --- | --- | --- | --- |
|  | Root | Stem | Leaves | Husk | Cob | Grain |
| 2013 |  |  |  |  |  |  |
| Silking | 18.23±0.29b | 20.28±0.92a | 23.55±0.32a | 21.28±0.98a | 19.25±0.87a | |
| 10DAS | 20.33±0.14a | 15.05±0.46b | 22.03±0.26ab | 11.18±0.34b | 8.33±0.60b | 20.00±0.48a |
| 20DAS | 19.28±0.69ab | 10.08±0.32c | 20.48±0.46bc | 8.48±0.34cd | 6.80±0.31b | 17.23±0.26c |
| 30DAS | 18.95±0.71ab | 9.13±0.58c | 19.15±0.82cd | 10.05±1.11bcd | 8.25±1.58b | 19.58±0.44ab |
| 40DAS | 19.38±0.48ab | 9.93±0.36c | 16.98±0.51d | 10.55±0.49bc | 7.10±0.33b | 18.83±0.16b |
| 50DAS | 17.58±0.85b | 9.70±0.63c | 14.53±1.48e | 8.03±0.70d | 6.50±0.45b | 18.60±0.30b |
| 2014 |  |  |  |  |  |  |
| Silking | 10.68±0.47c | 12.72±0.21a | 23.82±0.47a | 11.45±0.19a | 14.00±0.23a | |
| 10DAS | 14.58±0.28a | 9.39±0.59b | 20.97±0.77b | 8.52±0.29b | 11.25±0.92b | 19.08±0.47a |
| 20DAS | 13.16±0.36ab | 8.43±0.58b | 17.16±0.13c | 6.65±0.47c | 6.22±0.92c | 15.09±0.51c |
| 30DAS | 14.65±0.76a | 7.97±0.8bc | 12.85±0.27d | 5.80±0.67cd | 4.49±0.55cd | 15.84±0.41bc |
| 40DAS | 12.63±0.67b | 6.56±0.06cd | 13.73±0.26d | 5.15±0.83d | 4.63±0.34cd | 16.18±0.47bc |
| 50DAS | 13.52±0.70ab | 6.03±0.49d | 13.35±0.80d | 5.09±0.21d | 3.80±0.16d | 16.74±0.07b |
| Source of variance | | |  |  |  |  |
| Year(Y) | *** | *** | *** | *** | *** | *** |
| Sampling dates (S) | *** | *** | *** | *** | *** | *** |
| S*Y | * | *** | *** | *** | *** | * |

Data are means ± SE. Within columns, different letters indicate significant differences at P < 0.05 between different sampling dates in a year. ***, **, * indicate significance at 0.001, 0.01, 0.05 probability level, respectively. NS means not significant at the 0.05 probability level.
