# Supplementary material for: Use of the Stable Nitrogen Isotope to Reveal the Source-Sink Regulation of Nitrogen Uptake and Remobilization during Grain Filling Phase in Maize
Source: PLoS One. 2016 Sep 8;11(9):e0162201. doi: 10.1371/journal.pone.0162201 (PMC5015999; doi:10.1371/journal.pone.0162201)
Supplement: S3 Table — (DOC) [file pone.0162201.s004.doc]

**Supporting information:**

S3 Table. Dynamic changes of N content in different organs from silking to physiological maturity.

|  | N content(g plant-1) | |  |  |  |  |  |
| --- | --- | --- | --- | --- | --- | --- | --- |
|  | Root | Stem | Leaves | Husk | Cob | Grain | Whole plant |
| 2013 |  |  |  |  |  |  |  |
| Silking | 0.20±0.01ab | 0.63±0.01a | 0.46±0.02a | 0.09±0.00a | 0.06±0.00bc |  | 1.43±0.04d |
| 10DAS | 0.22±0.01a | 0.44±0.02b | 0.45±0.03a | 0.06±0.01b | 0.05±0.01c | 0.31±0.01e | 1.52±0.04d |
| 20DAS | 0.20±0.01ab | 0.36±0.01c | 0.49±0.02a | 0.05±0.00b | 0.06±0.00bc | 0.74±0.01d | 1.89±0.03c |
| 30DAS | 0.18±0.02ab | 0.31±0.02c | 0.43±0.02a | 0.05±0.01b | 0.11±0.02a | 1.05±0.01c | 2.11±0.05b |
| 40DAS | 0.18±0.01ab | 0.33±0.02c | 0.31±0.01b | 0.05±0.00b | 0.10±0.00a | 1.34±0.01b | 2.30±0.04a |
| 50DAS | 0.16±0.01b | 0.30±0.03c | 0.31±0.03b | 0.04±0.01b | 0.08±0.01b | 1.45±0.02a | 2.34±0.07a |
| 2014 |  |  |  |  |  |  |  |
| Silking | 0.13±0.00b | 0.41±0.00a | 0.59±0.01a | 0.08±0.00a | 0.13±0.00a |  | 1.33±0.01e |
| 10DAS | 0.18±0.01a | 0.36±0.02a | 0.62±0.01a | 0.08±0.00a | 0.13±0.01a | 0.17±0.02e | 1.54±0.03d |
| 20DAS | 0.11±0.00b | 0.35±0.03a | 0.50±0.01b | 0.07±0.01b | 0.08±0.01b | 0.52±0.03d | 1.62±0.05cd |
| 30DAS | 0.13±0.02b | 0.27±0.02b | 0.35±0.02c | 0.04±0.00c | 0.06±0.01bc | 0.86±0.03c | 1.71±0.03c |
| 40DAS | 0.12±0.01b | 0.23±0.00bc | 0.35±0.01c | 0.03±0.01c | 0.05±0.01bc | 1.11±0.02b | 1.89±0.04b |
| 50DAS | 0.13±0.01b | 0.21±0.01c | 0.36±0.03c | 0.04±0.00c | 0.05±0.00c | 1.29±0.02a | 2.09±0.01a |
| Source of variance | |  |  |  |  |  |  |
| Year(Y) | *** | *** | *** | NS | NS | *** | *** |
| Sampling dates (S) | *** | *** | *** | *** | ** | *** | *** |
| S*Y | NS | *** | *** | ** | *** | NS | *** |

Data are means ± SE. Within columns, different letters indicate significant differences at P < 0.05 between different sampling dates in a year. ***, **, * indicate significance at 0.001, 0.01, 0.05 probability level, respectively. NS means not significant at the 0.05 probability level.
